# DNA Damage-Inducing 10-Methoxy-canthin-6-one (Mtx-C) Promotes Cell Cycle Arrest in G_2_/M and Myeloid Differentiation of Acute Myeloid Leukemias and Leukemic Stem Cells

**DOI:** 10.1021/acsomega.4c05435

**Published:** 2024-08-22

**Authors:** Heron F. V. Torquato, Manoel Trindade Rodrigues Junior, Cauê Santos Lima, Roberto Theodoro de Araujo Júnior, Caio C. S. P. Soares, André Tarsis Domiciano, Rafael Leite Tavares de Morais, Daiane Rosolen, Luciane Regina Cavalli, Osvaldo Andrade Santos-Filho, Giselle Zenker Justo, Ronaldo Aloise Pilli, Edgar J. Paredes-Gamero

**Affiliations:** † Faculdade de Ciências Farmacêuticas, Alimentos e Nutrição, Universidade Federal de Mato Grosso do Sul, Campo Grande, MS 79070-900, Brazil; ‡ Departamento de Bioquímica, Universidade Federal de São Paulo, R. Três de Maio 100, São Paulo, SP 04044-020, Brazil; § Departamento de Biofísica, Universidade Federal de São Paulo, R. Três de Maio 100, São Paulo, SP 04044-020, Brazil; ∥ Instituto de Química, Universidade Estadual de Campinas, Campinas, SP 13084-971, Brazil; ⊥ Instituto de Pesquisa Pelé Pequeno Príncipe, Curitiba 80250-060, Brazil; # Lombardi Comprehensive Cancer Center, Department of Oncology, Georgetown University, Washington, D.C. 20007, United States; ∇ Laboratório de Modelagem Molecular e Biologia Estrutural Computacional, Instituto de Pesquisas de Produtos Naturais Walter Mors, Centro de Ciências da Saúde, Universidade Federal do Rio de Janeiro, Av. Carlos Chagas Filho, 373 - Bloco H, Cidade Universitária, Rio de Janeiro 21941-599, Brazil

## Abstract

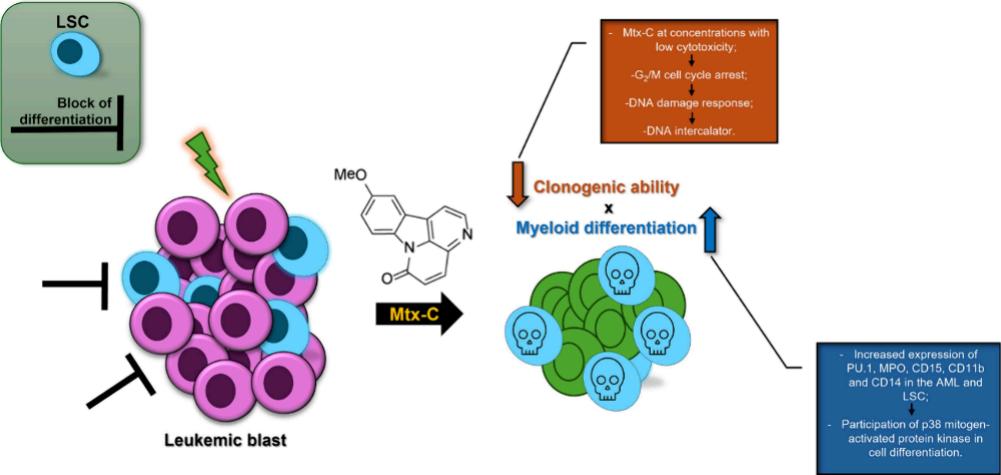

Synthetic 10-methoxy-canthin-6-one (Mtx-C), an alkaloid derivative, exhibits cytotoxic effects against acute myeloid cells (AMLs) and leukemic stem cells (LSCs) at a concentration of approximately 60 μM. However, the antitumor mechanism of Mtx-C in AMLs and LSCs remains elusive. Using Mtx-C at concentrations with low cytotoxicity (2–4 μM) for 72 h, we observed cell arrest with the accumulation of cells in the G_2_/M phase of the cell cycle. This effect was controlled by cyclin B1 expression and induction of the DNA damage cascade characterized by ATM, ATR, Chk1/2, p53, and H2A.X phosphorylation. Molecular docking analysis confirmed Mtx-C as a DNA intercalator. Moreover, the expression of inhibitors of cyclin-dependent kinases, including p21 (Cip1) and p27 (Kip1), increased. In addition, several miRNAs that are considered oncosuppressors were regulated by Mtx-C in Kasumi-1 cells. Finally, concomitant with cell cycle arrest, the underlying molecular mechanisms of Mtx-C in AML cells include myeloid differentiation, as evidenced by the increased expression of PU.1, myeloperoxidase, CD15, CD11b, and CD14 in the AML and LSC populations with the participation of p38 mitogen-activated protein kinase. Thus, we showed that Mtx-C simultaneously induced cell cycle arrest and myeloid differentiation in AML lineages and in the LSC population, providing insights into new therapeutic alternatives for the treatment of AML based on naturally occurring molecules.

## Introduction

Acute myeloid leukemia (AML) is a highly heterogeneous blood disorder with an increased incidence in older adults. Additionally, differentiation arrest at hematopoietic progenitor stages is a classical hallmark of myeloid malignancies, such as AML, in addition to severe pancytopenia, relapse, and failure of first-line therapy, which occur in a considerable proportion of patients.^1^ The major obstacle in the treatment of AML is the resistance of the clonal subset population known as leukemia stem cells (LSCs) to common chemotherapy, which promotes AML relapse.^1,2^ LSCs exhibit stem cell features such as stem cell marker expression, self-renewal, and quiescence.^1,2^

Canthin-6-one and Mtx-C (10-methoxy-6*H*-indolo[3,2,1-de][1,5]naphthyiridin-6-one), a canthin-6-one analog, exhibit promising characteristics, positioning them as excellent candidates for ongoing efforts in optimizing the design of antileukemic drugs. Notably, canthin-6-one is a subclass of carboline alkaloids with an additional D-ring^3^ as noted in various pharmacological and toxicological studies,^4^ and its antiviral properties have been tested against HIV,^5^ and Parkinson disease due to its ability to induce the degradation of alpha-synuclein (α-syn).^6^ Other studies showed that canthin-6-one obtained from the stem bark of *Ailanthus altissima* inhibited the lipopolysaccharide-induced expression of inducible nitric oxide synthase and other inflammatory markers, especially the transcriptional activation of nuclear factor kappa B, in macrophages.^7^ As an antiparasitic agents, the antileishmanial activity of *Zanthoxylum chiloperone* extract is attributed to the presence of canthin-6-one and 5-methoxycanthin-6-one.^8^

In a previous report, we explored the effects of high concentrations of canthin-6-one and Mtx-C on the cell death mechanism in leukemic cells and leukemia patient samples (∼60 μM). In particular, the underlying molecular mechanisms of Mtx-C include apoptotic and necroptotic activation, the induction of stress signaling pathways involving mitogen-activated protein kinases (MAPKs) p38 and c-Jun, and DNA damage.^9,10^ Herein, we explored the effect of Mtx-C on the proliferation and myeloid differentiation of AML cells and primitive subsets of LSCs using concentrations below the half-maximal effective concentration (EC_50_) with a proliferation assay and determined that Mtx-C has low cytotoxicity. We confirmed that Mtx-C induced cell cycle arrest by binding to DNA and modulating DNA damage response (DDR) signaling pathways, promoting the differentiation of AML and LSC populations.

## Results

### Low Mtx-C Concentrations Reduce the Clonal Capacity of AML Cell Lines Due to DNA Damage

In our previous investigation, we reported the cytotoxicity of canthin-6-one and its derivatives in AML cells, with EC_50_ values ranging from 36 to 80 μM after 24 h of treatment.^9,10^ Herein, a comprehensive examination of the anticancer effects of Kasumi-1 and KG-1 on human AML lineages was performed using concentrations with low cytotoxic potential. These cell lines were exposed to repeated concentrations of Mtx-C below the EC_50_ value. The impact on cell proliferation was analyzed for 24 to 72 h.

**Figure 1:**
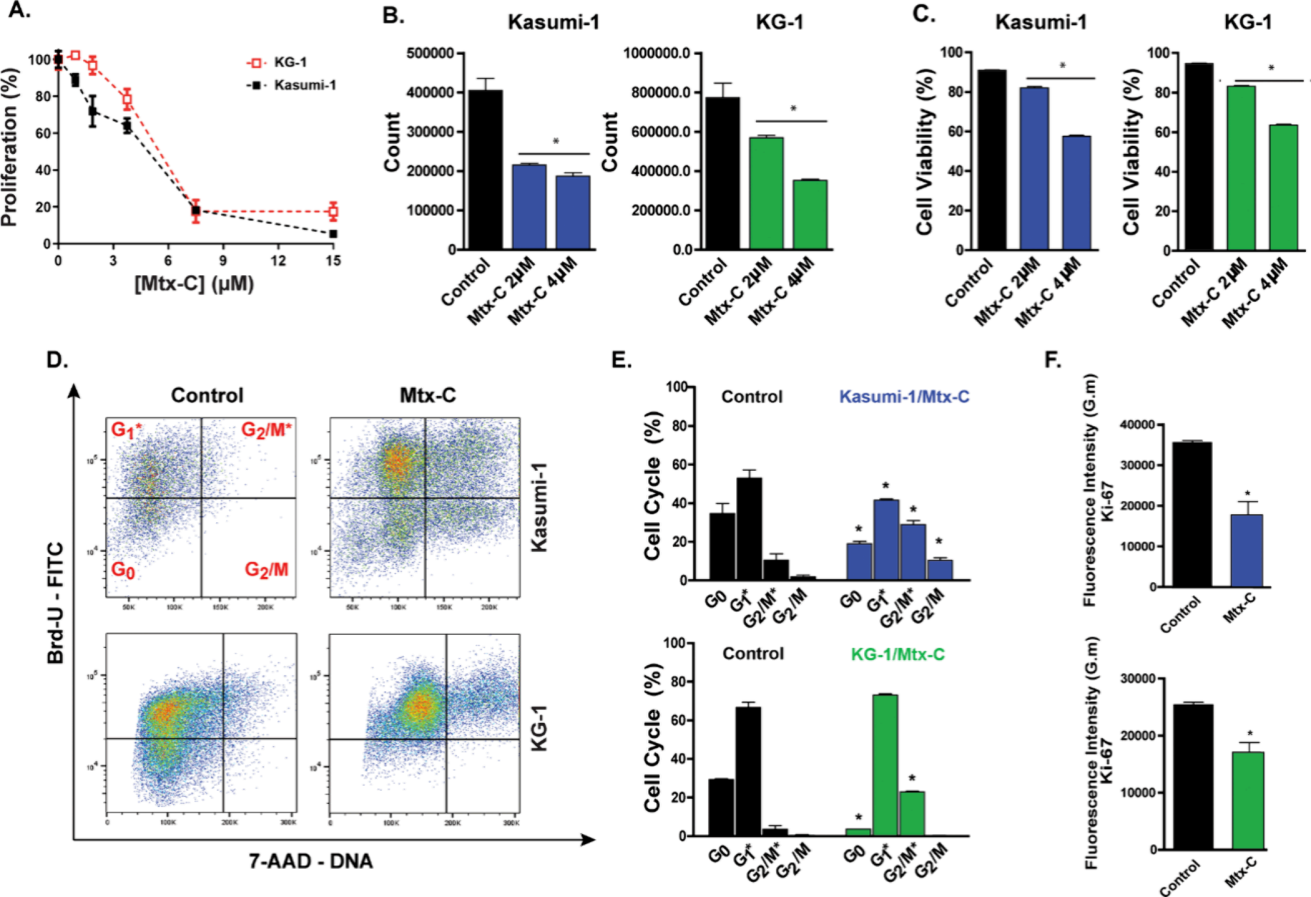
**Mtx-C decreased cell proliferation and induced G**_**2**_**/M cell cycle arrest in human AML cell lines.** Kasumi-1 and KG-1 cells were stimulated once daily for 72 h with different concentrations (15, 7.5, 3.7, 1.8, and 0.9 μM) or 2 or 4 μM Mtx-C. (A) Brd-U incorporation into DNA was detected by absorbance. (B) Cell counting. (C) Cell viability was measured using annexin V-FITC and 7-AAD staining. (D-E) BrdU incorporation and DNA content analysis using flow cytometry. (F) *K*_i_-67 expression. The data represent the mean ± SEM *p*<*0.05. Student’s *t* test or one-way ANOVA followed by Dunnett’s post hoc test was used.

Initially, we determined the EC_50_ value for Mtx-C using the Brd-U proliferation assay. EC_50_ values were calculated using different Mtx-C concentrations (15, 7.5, 3.7, 1.8, and 0.9 μM) applied once daily for 72 h. Our results showed an EC_50_ of 5.1 ± 1.6 μM for Kasumi-1 cells and 6.0 ± 2.2 μM for KG-1 cells (Figure 1A).

Then, the effects of Mtx-C on cell counts and cell death were assessed using flow cytometry. Both cell lines were exposed to Mtx-C concentrations of 2 μM and 4 μM for 24 h (one application), 48 h (two applications) (Figure S1A-D) and 72 h (three applications) (Figure 1B, C). A reduction in cell count was observed during the experiment at both concentrations, but cell death was noted in less than 20% of Kasumi-1 cells and 40% of KG-1 cells (Figure 1B, C).

For all subsequent experiments, Kasumi-1 and KG-1 cells were treated with 2 μM and 4 μM Mtx-C, respectively, three times over a 72 h period. Dot plots obtained from Brd-U incorporation and DNA staining using flow cytometry showed a significant reduction in G_0_, an increase in the G_2_/M phase of the cell cycle (Figure 1D, E), and a decrease in the expression of *K*_i_-67 (Figure 1F), a protein expressed during active phases of the cell cycle.

**Figure 2:**
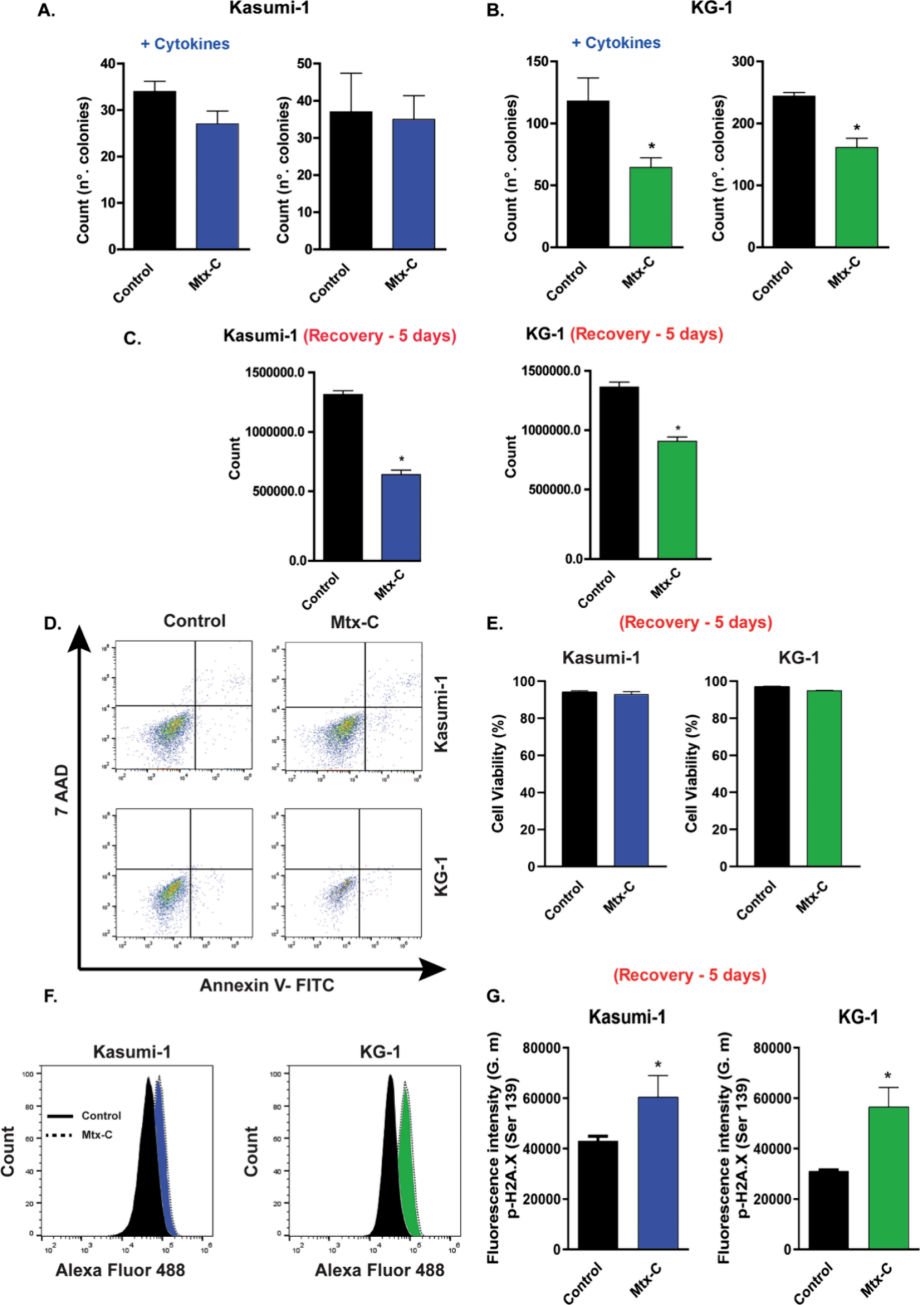
**Mtx-C reduced the colony growth and recovery potential of AML cell lines.** Kasumi-1 and KG-1 cells were stimulated once daily for 72 h with 2 or 4 μM Mtx-C, respectively. After stimulation, the cells were seeded on MethoCult H4100 (without cytokines) or MethoCult H4434 (with cytokines) (A-B), or replated in new cell culture medium for 5 d for counting (C). (D-E) Cell viability after recovery for 5 d assessed by annexin V-FITC and 7-AAD staining. (F-G) Quantification of H2A.X phosphorylation. The results are presented as the means ± S.E.M.s of three independent experiments performed in triplicate. Student’s *t* test. *p < 0.05 versus control.

In addition, colony forming assays performed in methylcellulose-based medium with and without recombinant cytokines indicated that Mtx-C treatment resulted in a significant decrease in colony-forming units (CFUs) for KG-1 cells but not for Kasumi-1 cells (Figure 2A, B).

To evaluate the extent of cell recovery after Mtx-C treatment for 72 h, Kasumi-1 and KG-1 cell lines were cultivated for more than **5 d** in fresh liquid culture media without the addition of Mtx-C. Treatment with Mtx-C decreased cell recovery (Figure 2C) without causing a loss of viability (Figure 2D, E). However, cells exhibited persistent DNA damage signaling, as determined by increased phospho-H2A.X (Figure 2F, G). Furthermore, Mtx-C did not promote cytotoxicity or H2A. X phosphorylation in peripheral blood mononuclear cells (PBMCs) after 72 h of treatment. (Figure S2 A-D).

### Molecular Docking Approach Reveals Differences in the Binding of Mtx-C and Canthin-6-one to DNA

**Figure 3:**
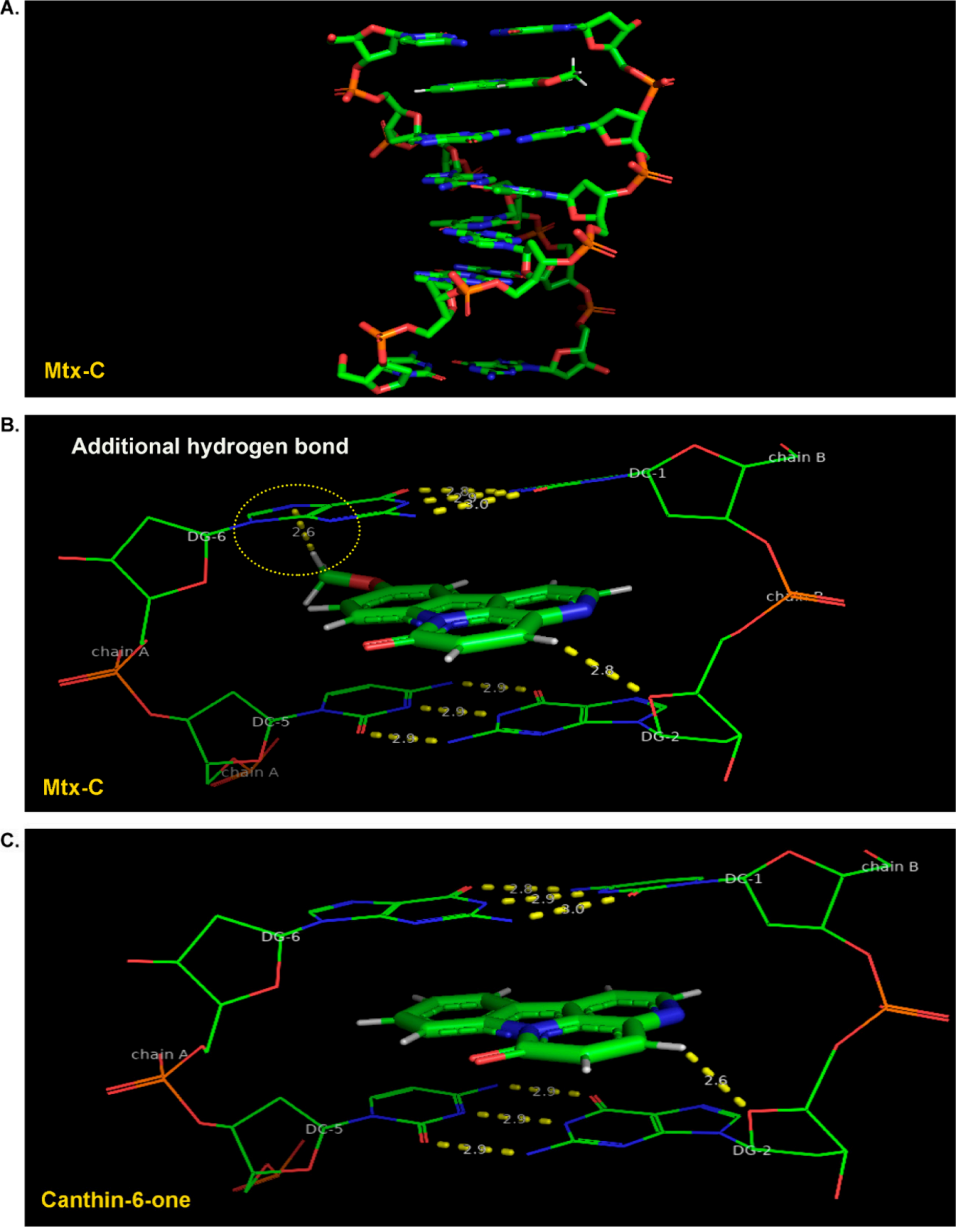
**Molecular docking of Mtx-C to the DNA oligomer d(CGATCG)**_**2**_**.** (A) 3D representations of 10-methoxycanthin-6-one docked with DNA. (B) Nonbonded interactions of the macromolecular DNA/10-methocycanthin-6-one complex. (C) Nonbonded interactions of the macromolecular DNA/canthin-6-one complex.

To verify how canthin-6-one and Mtx-C interact with DNA, a molecular docking simulation was performed. Structural analysis of the generated macromolecular complexes revealed that both compounds intercalate parallel to the hydrogen bonds of the base pairs and stack their aromatic rings into CG bases of DNA. Figure 3A shows a modeled 3D structure of Mtx-C docked with DNA. Figures 3B and 3C depict detailed views of the docked poses of Mtx-C and canthin-6-one. The calculated intermolecular energies (affinity) for Mtx-C and canthin-6-one were −7.5 and −7.3 kcal/mol, respectively. The difference in energy values suggests that Mtx-C has a slightly stronger interaction with DNA than canthin-6-one. As shown in Figure 3B and Figure 3C, the “extra” binding stabilization of Mtx-C is due to the additional hydrogen bond between the methoxy group and the imidazole ring of G6 of DNA.

### Cell cycle arrest induced by Mtx-C is DNA damage dependent

**Figure 4:**
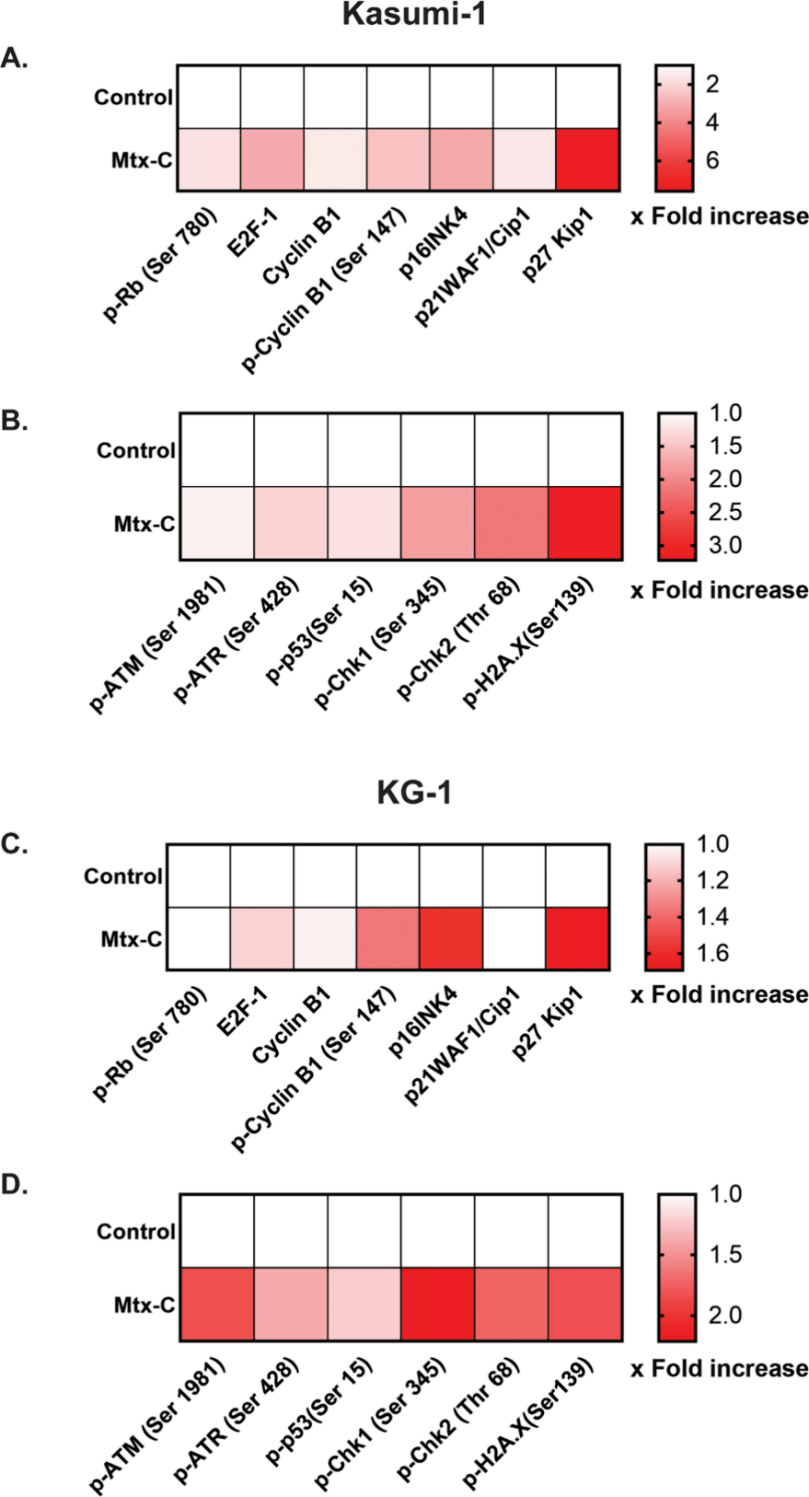
**Proteins associated with G**_**2**_**/M cell cycle arrest induced by Mtx-C in human AML cell lines.** Kasumi-1 and KG-1 cells were stimulated once daily with 2 or 4 μM Mtx-C, respectively, for 72 h. Protein expression and phosphorylation in (A-B) Kasumi-1 or (C–D) KG-1 cells were analyzed using flow cytometry. The fold increase in the normalized results is shown in a graphical heatmap of the geometric mean fluorescence intensity. The data are presented as the means ± S.E.M.s.

Molecular docking predicted a chemical interaction between Mtx-C and DNA. Cell cycle arrest and increased phospho-H2A.X were observed after treatment with Mtx-C. We further investigated the modulation of the retinoblastoma protein (Rb), E2F-1, cyclin B1, and cyclin-dependent kinase inhibitors (CDKIs) p16 (INK4a), p21 (Cip1), and p27 (Kip1) by Mtx-C. A heatmap based on fluorescence intensity showed an increase in Rb protein phosphorylation after treatment in both cell lines (Figure 4A and C). Cyclin B1 protein levels were detected only in Kasumi-1 cells (Figure 4A), although cyclin B1 phosphorylation was observed in both cell lines (Figure 4A and C). Increased levels of the proteins p16, p21, and p27 in Kasumi-1 cells and of p21 and p27 (Figure 4A and C) in KG-1 cells were observed after Mtx-C treatment.

The results thus far indicate convergent cell cycle arrest and Mtx-C interaction with DNA, as demonstrated by molecular docking. The DDR promoted by Mtx-C leads to cell cycle checkpoint activation and blockade of cell cycle progression.^11^ Thus, we investigated the phosphorylation of the DNA-responsive proteins ataxia-telangiectasia mutated (ATM), ATM Rad3 related (ATR), checkpoint kinases 1 and 2 (ChK1 and Chk2), p53 and histone H2A.X in KG-1 and Kasumi-1 cells. Mtx-C increased the phosphorylation levels of all proteins evaluated (Figure 4B and D), except for ATM, in Kasumi-1 cells (Figure 4B). Non-normalized data are also presented with the geometric mean fluorescence intensity (Figure S4A-B).

### Mtx-C Alters microRNA Expression in Kasumi-1 Cells

**Figure 5:**
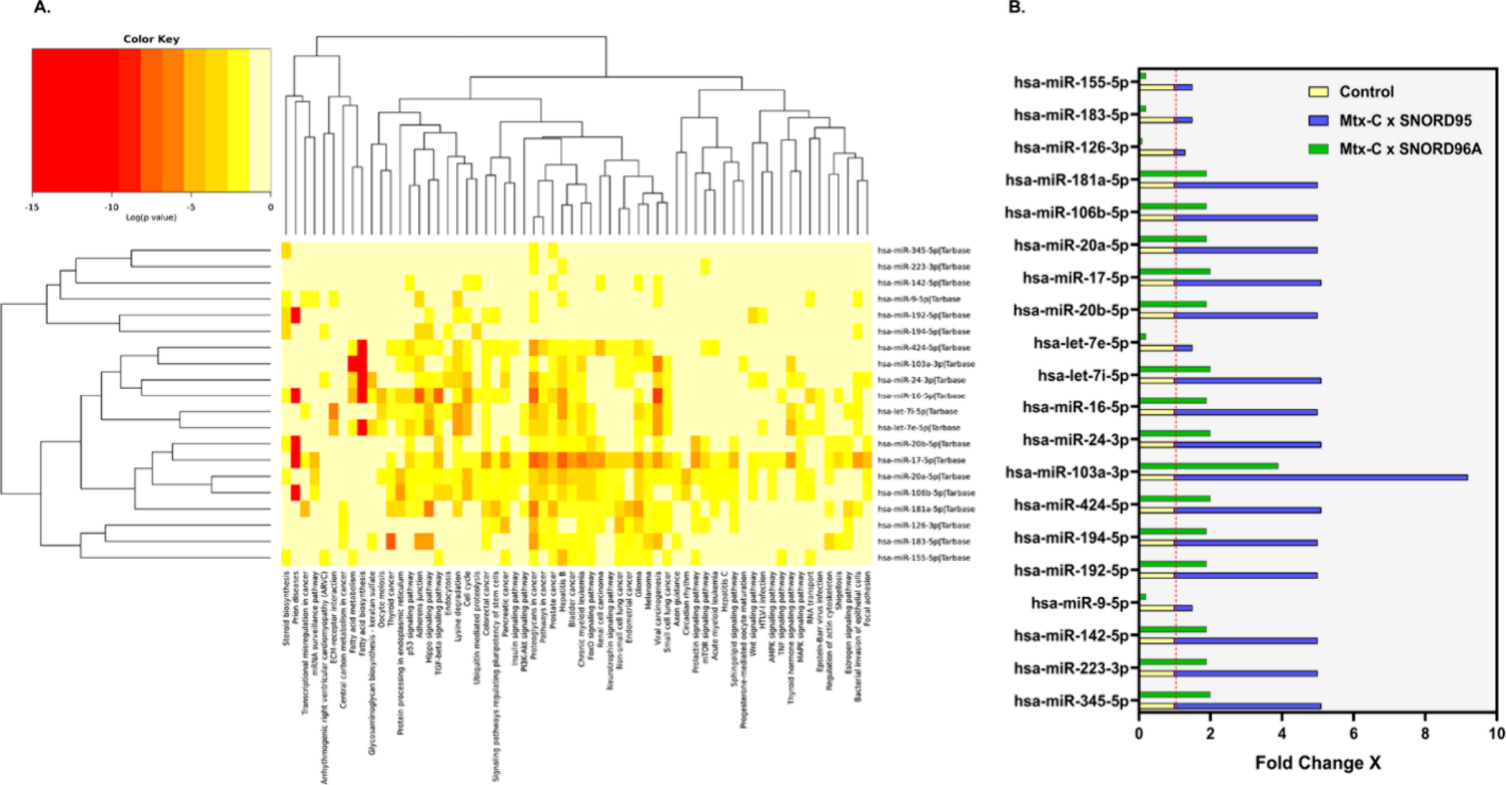
**Analysis of Kasumi-1 miRNA expression in response to Mtx-C treatment.** (A) Hierarchical clustering heatmap showing pathways related to these target genes or diseases. The colors and intensities indicate the expression level. Red indicates downregulated genes, and yellow indicates upregulated genes. (B) Differential expression results of the 20 miRNAs. microRNA quantification was evaluated based on the fold change and calculated using the 2^–ΔΔCT^ equation. SNORD95 and SNORD96A were selected by RefFinder as reference genes for analysis between the treated and control groups.

To further explore the mechanisms underlying the effects of Mtx-C on Kasumi-1 cells, we performed quantitative real-time PCR (RT–PCR) to detect and quantify the expression of miRNAs, and functional analysis of the results was performed using bioinformatic tools. Figures 5A and 5B depict the corresponding heatmap constructed from a hierarchical cluster analysis of the 20 miRNAs significantly regulated by Mtx-C (out of 84 miRNAs analyzed based on significance levels) and different pathways. Darker colors represent lower values of significance. Moreover, the genes targeted by the 11 differentially expressed miRNAs associated with cell death and proliferation and relevant signaling pathways were assigned using the Kyoto Encyclopedia of Genes and Genomes (KEGG) (Figure S3). According to the analysis, 27 related intracellular pathways were obtained with a standard threshold of *p* < 0.05.

### Mtx-C Induces Myeloid Differentiation in Kasumi-1 and KG-1 Lineages and Leukemia Stem Cell Subsets

**Figure 6:**
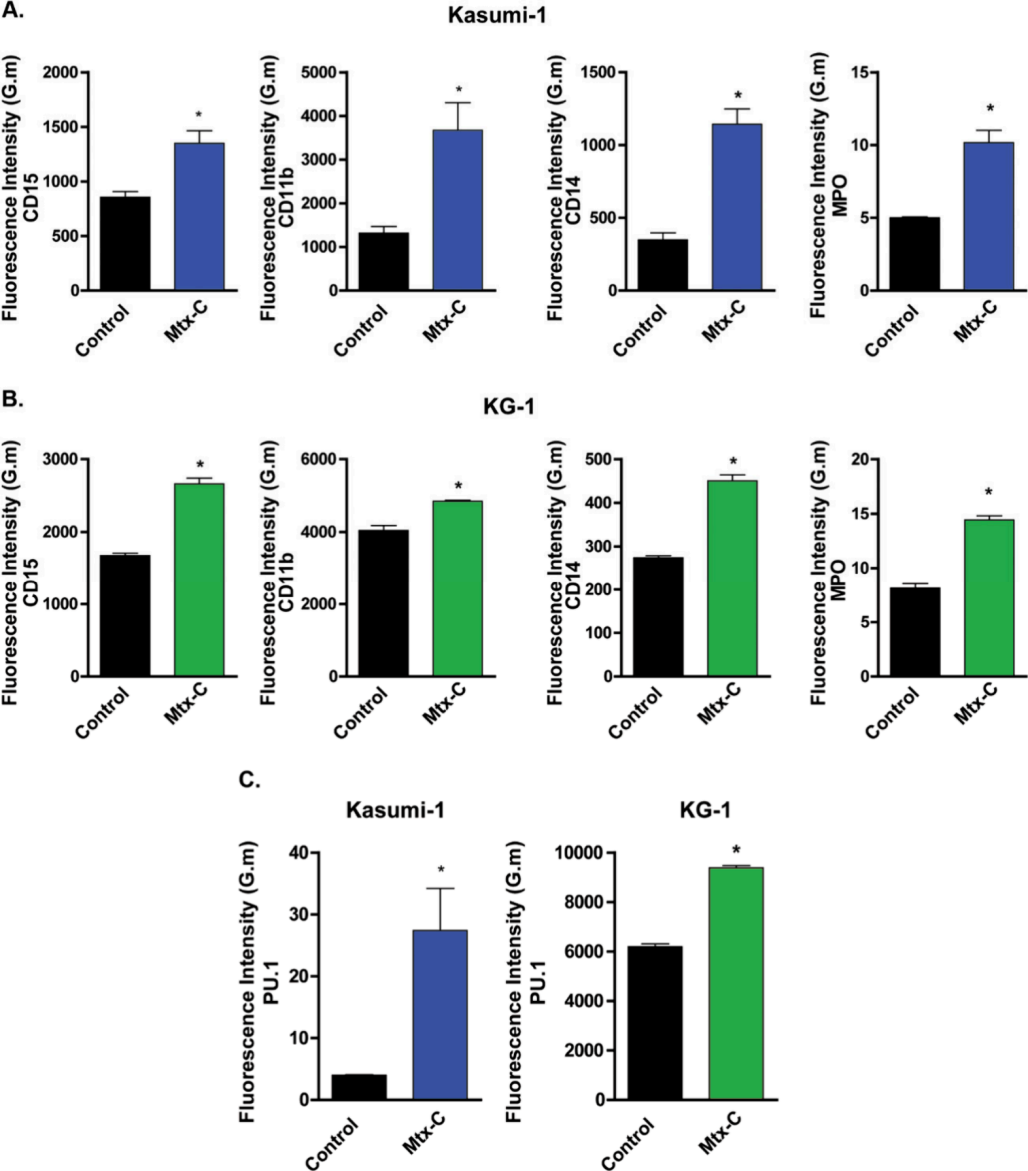
**Mtx-C induced an increase in myeloid marker expression in human AML cell lines.** Kasumi-1 (A) and KG-1 (B) cell lines were stimulated once daily with 2 or 4 μM Mtx-C, respectively, for 72 h. The expression of the markers of commitment myeloid CD15, CD11b, and CD14 and MPO increased after treatment with Mtx-C. (C) Increased expression of PU.1, a myeloid lineage-specific transcription factor, was also observed. The results are presented as the means ± S.E.M.s of 3 independent experiments performed in triplicate. Student́s *t* test. *p < 0.05 versus control.

Due to the importance of eliminating the LSC population in AML, the expression of myeloid markers associated with differentiation, such as myeloperoxidase (MPO), CD15, CD11b, CD14, and the transcription factor PU.1, was analyzed. We observed a significant increase the expression levels of these proteins after Mtx-C treatment in both cell lines (Figure 6A-C).

**Figure 7:**
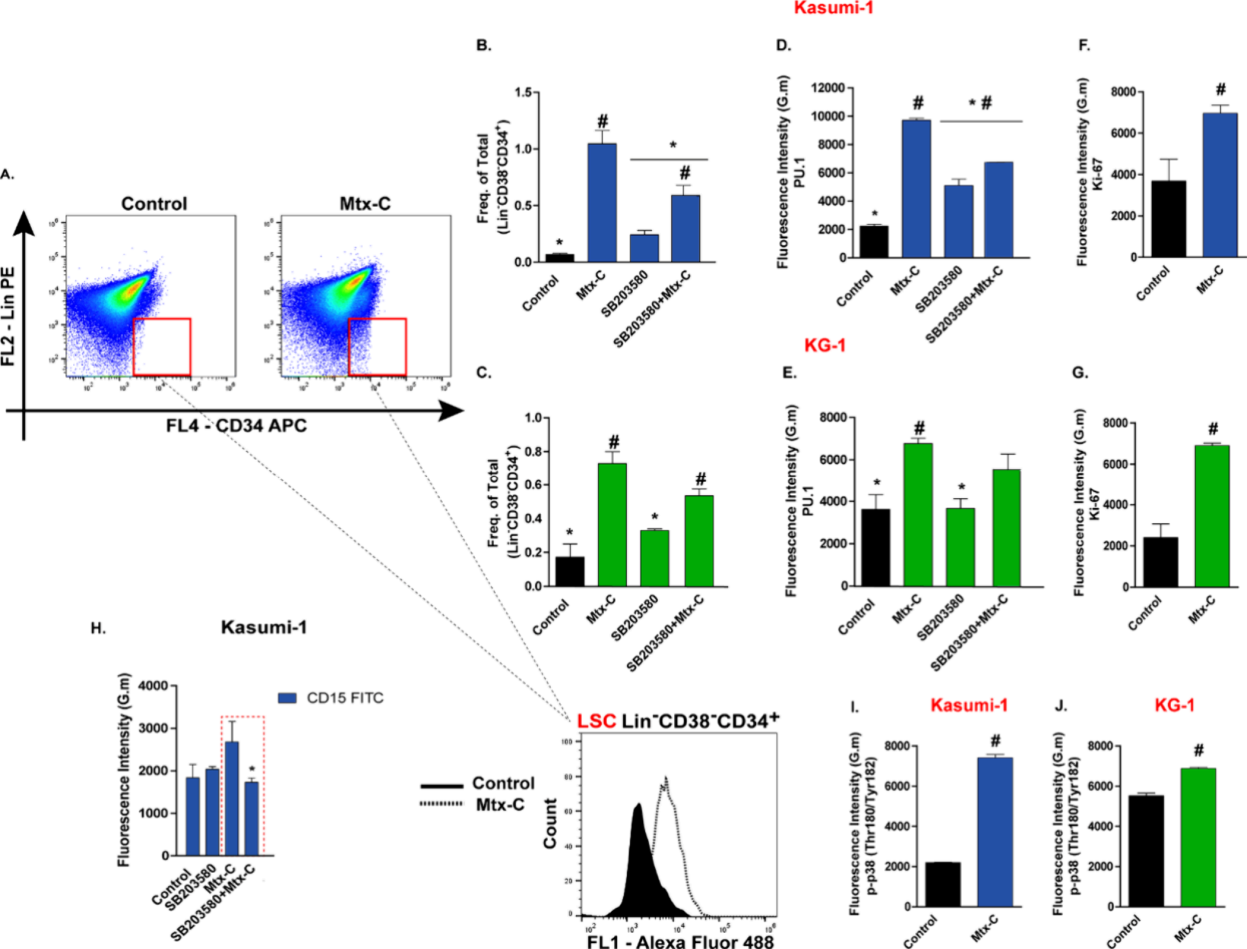
**LSC proliferation and differentiation were also affected by Mtx-C**. Kasumi-1 and KG-1 cells were stimulated once daily with 2 or 4 μM Mtx-C, respectively, for 72 h, and the number of LSCs was evaluated. (A) Dot plots from flow cytometry analysis. (B–C) Frequency of the LSC population. (D-E) PU.1 protein expression in Kasumi-1 and KG-1 cells treated with or without SB203580 (20 μM). (F-G) *K*_i_-67 expression. (H) SB203580 inhibited the increase in the myeloid marker CD15 in Kasumi-1 cells treated with Mtx-C. (I-J) Quantification of cytometry histograms showing p38 MAPK phosphorylation. The results are presented as the means ± S.E.M.s of 3 independent experiments performed in triplicate. One-way ANOVA followed by Dunnett’s test; *p < 0.05 versus Mtx-C, # *p* < 0.05 versus control. Student’s *t* test. ^#^*p* < 0.05 versus control.

Furthermore, the differentiation potential of Mtx-C in LSCs was also investigated. Identification of the LSC population in the Kasumi-1 and KG-1 lineages (Lin^–^CD34^+^CD38^–^) is shown in Figure 7A. Treatment with Mtx-C increased the frequency of LSCs (Figure 7B and C) and the expression of PU.1 (Figure 7D and E) and *K*_i_-67 (Figure 7F and G).

Previously, we demonstrated the involvement of the p38 MAP kinase pathway in the cell death mechanisms induced by Mtx-C.^10^ To explore the mechanisms underlying the differentiation of LSCs triggered by Mtx-C, SB203580, a p38 MAP kinase inhibitor, was used. We observed reductions in Mtx-C effects, such as increases in the frequency of LSCs in both lineages and increases in PU.1 expression in the Kasumi-1 lineage (Figure 7B-E). Preincubation of Kasumi-1 cells with SB203580 partially reduced the increase in CD15 (Figure 7H), and an increase in p38 MAPK phosphorylation was observed in both cell lines, corroborating the key role of Mtx-C in cell differentiation (Figure 7I and J).

## Discussion

Diverse natural and synthetically derived β-carboline alkaloids have shown promising inhibitory effects on the growth of cancer cells and have served as a starting point for drug design.^12^ These effects are consistent with data obtained from previous reports of canthin-6-one and Mtx-C in AML cell lines,^9,10^ human prostate cancer cells,^13^ and recently, canthin-6-one derivatives with *N*-methylpiperazine substitution exhibited high cytotoxicity against HT29, A549 and MCF-7 cells.^14^

Based on these observations, we investigated the molecular mechanisms related to cell cycle arrest associated with DNA damage and the potential of Mtx-C to induce myeloid differentiation in AML cell lines and the LSC subset using concentrations below the EC_50_ in assessments of cytotoxicity and close to the EC_50_ to study proliferation inhibition.

Our data revealed that cell accumulation in the G_2_/M phase was controlled by cyclin B1 expression and phosphorylation and ATM, ATR, Chk1/2, p53 and H2A.X phosphorylation. In addition to molecular docking studies, which revealed the ability of Mtx-C to intercalate DNA (Figure 3A-B), DNA damage signaling was activated by Mtx-C (Figure 4A-D). Moreover, the expression of CKIs increased (Figure 4A and C). These proteins belong to a family of cell cycle regulators and form stable complexes with cyclin-dependent kinases, subsequently inhibiting cell proliferation.^15^ Thus, the effects of Mtx-C are dependent on DNA damage and the consequent arrest in the G_2_/M cell cycle phase. These effects are similar to the effects of cytotoxic drugs such as Temozolomide, which promotes cell cycle arrest by activating ATM/ATR, p53 and Chk1/2 signaling in lymphoblastoid cells;^16^ ICRF-193, a topoisomerase II inhibitor that results in a greater proportion of cells arrested in the G_2_ phase, concurrently with increased p53 and p21 levels;^17^ and carfilzomib, a proteasome inhibitor that results in significant inhibition of endometrial tumor cell proliferation and increased p21 and p27 levels.^18^ Interestingly, harmine, a β-carboline alkaloid, is an efficient Cdk inhibitor, especially with regard to Cdk2 and 5.^19^ Additionally, our results indicate that Mtx-C increased p16 protein expression, even in G_2_/M arrest. A similar result was found for oridonin, a diterpene isolated from *Rabdosia rubescens*. This study showed that oridonin induced cell cycle arrest at the G_2_/M phase, increasing p16, p21 and p27 expression in colorectal cancer cells.^20^

Additionally, our study examined the role of miRNAs that have been identified as oncogenes or oncosuppressors, and their expression is associated with the development of cancers, including hematological malignancies. Consistent with the aforementioned findings, many of these miRNA targets are known to be cell cycle and cell death regulators. Of these, the miR-17–92 cluster, led by its most prominent member, miR-17–5p, comprises some of the most well studied thus far. Although this miRNA is considered an oncogene, its functions in suppressing metastasis and activating T cells have been described in the literature.^21,22^ Interestingly, upregulation of miR-20a was associated with a greater complete remission rate and longer overall survival in patients with AML.^23^ In addition, studies have indicated important roles for miR-16–5p in carcinogenesis, and the first evidence of its tumor suppressor functions was obtained in chronic lymphocytic leukemia.^24^ In murine erythroleukemia, miR-16–5p plays a role in promoting erythroid differentiation.^25^ The expression of miR-155–5p has been observed in diffuse large B-cell lymphoma, AML, and chronic lymphocytic leukemia.^26^ In AML patients, this miR was considered an unfavorable prognostic factor, and its deregulation was associated with a profile enriched for genes involved in inflammation and apoptosis.^27,28^ Although this study revealed that other miRNAs were significantly altered by Mtx-C treatment, further studies are needed to establish a functional correlation between these miRNAs and their targets.

Proliferation of hematopoietic stem cell populations or LSCs is associated with self-renewal or differentiation.^9,29,30^ Interestingly, Mtx-C induced cell differentiation as evidenced by the increased expression of PU.1, MPO, CD15, CD11b and CD14. An important feature of AML is its deficiency in mechanisms that cause cell differentiation.^31^ This may represent greater susceptibility to infections, hemorrhages or anemia since the individual will have a smaller number of functional cells of the hematopoietic system.^32^

Therapies that use cell differentiation as a strategy for leukemia treatment date back to the 1970s and 1980s, and cell differentiation is an interesting alternative to therapies that only induce cytotoxicity.^33^ The most notable success was the discovery of all-trans-retinoic acid (ATRA) used to treat acute promyelocytic leukemia.^34^ After the introduction of ATRA in combination with standard chemotherapy, the prognosis of acute promyelocytic leukemia patients improved substantially, with greater than 85% of patients achieving disease remission and approximately 70% being cured.^35^ Other differentiation-based therapies include histone deacetylase inhibitors, such as valproic acid, which promote apoptosis and differentiation in different leukemia lines, including Kasumi-1 cells and patient samples.^36,37^ These examples collectively point to an alternative pathway in the treatment of patients with AML, demonstrating its efficacy in achieving higher rates of disease remission and cure.

We observed an increase in the percentage of LSCs and *K*_i_-67 but a reduction in clonogenic capacity and cell cycle arrest after 72 h of Mtx-C treatment. As previously observed in other hematopoietic stem cells or LSCs, the initial stimulus produces transient proliferation strongly associated with differentiation.^9,30,38^ The importance of investigating the effects of focus on LSCs is because most treatments do not reach these populations, which can again repopulate the tumor and lead to relapses.^39^ The use of Mtx-C in regimens combined with standard induction therapy could be an interesting strategy for investigating murine models and cells from AML patients.

Among the investigated mechanisms, preincubation with SB203580, a p38 MAPK inhibitor, partially reversed the effects on LSC differentiation and proliferation. An increase in p38 MAPK phosphorylation was observed after treatment. In leukemic cells, activation of p38 MAPK with anisomycin promoted senescence and apoptosis in LSCs from the K-562 and KG-1 cell lines.^40^ These findings were associated with the accumulation of oxidative DNA damage. Another important discovery was reported in a study demonstrating that umbilical cord mesenchymal cells incubated with HL60 and K-562 leukemic cells increased p38 MAPK phosphorylation, thus inhibiting leukemic cell growth.^41^ In cancer stem cells isolated from the lung, p38 MAPK inactivation contributes to the maintenance of this population in a more undifferentiated state.^42^ All these studies indicate that activated p38 MAPK functions as a growth suppressor, highlighting its potential as a target for antitumor therapies to eliminate LSCs and other types of cancer stem cells.

## Conclusion

Based on these findings, we demonstrated that Mtx-C promoted cell cycle arrest after treatment with a low Mtx-C concentration for 72 h, leading to the accumulation of cells at the G_2_ to M phase and an increase in the levels of different cyclins and CKIs, which act as tumor suppressors associated with DDR. In addition, Mtx-C promotes the differentiation of AML and LSC subsets. Using multiple methods and molecular docking, we found that Mtx-C potentially represents a great scaffold for the design optimization of antileukemic drugs. This study represents a promising tool for the development of new antileukemic molecules and revealed the target for Mtx-C in AML cells, providing new insights into its anticancer mechanisms.

## Methods

### 10-Methoxy-canthin-6-one

Mtx-C (Figure.S5) was synthesized as previously described.^10^ Stock solutions of all compounds were prepared in dimethyl sulfoxide (DMSO), stored at −20 °C and diluted in culture medium before use. The final concentration of DMSO in the culture medium at any time was not greater than 0.1%.

### Cell Cultures

The human leukemia cell lines Kasumi-1 and KG-1 were obtained from the American Type Culture Collection (ATCC). KG-1 cells were maintained in Iscove’s modified Dulbecco’s medium (IMDM) supplemented with 20% fetal bovine serum (FBS) (Cultilab, Brazil), and Kasumi-1 cells were maintained in Roswell Park Memorial Institute (RPMI 1640) (Sigma-Aldrich, Germany) medium supplemented with 10% FBS. All cells were cultured in a medium supplemented with 100 U/mL penicillin (Sigma–Aldrich, Germany) and 100 μg/mL streptomycin (Sigma–Aldrich, Germany) in a humidified incubator containing 5% CO_2_ at 37 °C.

### Peripheral Blood Mononuclear Cell Isolation

PBMCs were obtained from three healthy donors. Human monocytes from healthy donors were collected after informed consent was obtained from the patients. The separation of mononuclear cells was performed with gradient centrifugation methods using Ficoll Histopaque-1077 (1.077 g/cm^3^) (Sigma–Aldrich, Germany) following the manufacturer’s instructions. The use of human samples was approved by the local Ethical Committee of the Universidade Federal de Mato Grosso do Sul (CAAE35853720.2.0000.0021). The cells were maintained in IMDM supplemented with 20% FBS, 100 U/mL penicillin, and 100 μg/mL streptomycin in a humidified atmosphere at 37 °C in 5% CO_2_.

### BrdU Assay

Kasumi-1 and KG-1 cells (10^4^/mL) were treated once daily with different concentrations (15, 7.5, 3.7, 1.8, and 0.9 μM) of Mtx-C for 72 h. Then, cell proliferation analyses were performed using the Brd-U Cell Proliferation Assay #6813 Kit (Cell Signaling, USA), and the experiment was performed on a FlexSation 3 plate reader (Molecular Devices, USA).

For analysis of proliferation using flow cytometry, Kasumi-1 and KG-1 cells (10^5^/mL) were treated once daily with Mtx-C for 72 h in the presence of 10 μM BrdU (Sigma–Aldrich, Germany). Brd-U labeling was performed according to the manufacturer’s instructions (Brd-U-FITC Flow Kit, Becton Dickinson, USA). DNA content was labeled using 7-AAD (Becton Dickinson, USA). Data acquisition was performed using an Accuri C6 flow cytometer, and 100,000 events were acquired.

### Clonogenic Assay

Kasumi-1 or KG-1 (10^5^/mL) cells were treated with 2 μM or 4 μM Mtx-C for 72 h, respectively. Then, 10,000 cells were mixed with methylcellulose-based medium (MethoCult H4100) or methylcellulose-based medium supplemented with recombinant cytokines for human cells (MethoCult H4434): Stem cell factor (SCF), interleukin 3 (IL-3), erythropoietin (EPO), granulocyte-macrophage colony-stimulating factor (GM-CSF) (Stem Cell Technologies, USA). The mixture was placed in 35 mm dishes and cultured in a humidified incubator for 14 d. At the end of this period, colonies consisting of N50 cells were counted using an inverted microscope at 40× magnification.

### Annexin V/7-AAD Flow Cytometry Assay and Cell Counting

An Annexin V-FITC/7-AAD (7-aminoactinomycin D, Becton Dickinson, USA) double-staining assay was performed to evaluate the effect of Mtx-C on cell death. The cells were seeded (10^5^ cells/mL) in 6-well plates, and Kasumi-1 and KG-1 (10^5^/mL) cells were treated once daily with 2 μM or 4 μM Mtx-C for 72 h, respectively. Then, the cells were resuspended in annexin binding buffer (0.14 M NaCl, 2.5 mM CaCl_2_, 0.01 M HEPES, pH 7.4) and incubated at room temperature with 1 and 2 μL of annexin V-FITC and 7-AAD, respectively (Becton Dickinson, USA), for 30 min. The analysis was performed using an Accuri C6 flow cytometer and FlowJo software (Becton Dickinson, USA). A total of 10,000 events were collected per sample. Additionally, cell counting was performed automatically using an Accuri C6 flow cytometer (Becton Dickinson, USA).

### Intracellular Protein Labeling

Kasumi-1 and KG-1 cells (10^5^/mL) were treated once daily for 72 h with 2 μM or 4 μM Mtx-C, respectively. Then, the cells were fixed with BD Cytofix (Becton Dickinson, USA) for 15 min, washed with BD Perm/Wash buffer and permeabilized with Perm Buffer III (Becton Dickinson, USA) for 30 min at room temperature. To label intracellular proteins, the cells were incubated for 1 h with primary antibodies (see Table 1). The sections were then incubated with anti-rabbit or mouse IgG secondary antibodies conjugated with Alexa Fluor 488 (Thermo Fisher Scientific, USA) for at least 40 min, after which fluorescence was measured using an Accuri C6 flow cytometer and FlowJo software v.10 (Becton Dickinson, USA). A total of 40,000 events were collected per sample. Protein analyses were performed by quantifying the geometric mean (G.m).

### Assessment of Cell Differentiation Using Immunophenotyping

Kasumi-1 and KG-1 cells (10^5^/mL) were treated once daily for 72 h with 2 μM or 4 μM Mtx-C, respectively, once daily. Then, the cells were collected and stained to identify mature cells and leukemic stem cells. The LSC markers used included CD34-APC, CD38-PE and lineage (Lin) PE (CD2, CD3, CD4, CD7, CD8, CD14, CD19, CD20, and CD235a). CD15-FITC, CD11b-Cy7/PE, CD14-APC or MPO-FITC were also used in some experiments. All antibodies were purchased from Becton Dickinson (USA). The measurements were performed using an Accuri C6 flow cytometer (Becton Dickinson, USA). A total of 300,000 events were acquired.

### Molecular Docking Simulation of Mtx-C with B-DNA

The crystallographic structure of d(CGATCG)_2_ (PDB ID: 1Z3F)^43^ was used as the biomacromolecular receptor in molecular docking simulations. The three-dimensional structures of canthin-6-one and Mtx-C were obtained from PubChem. Molecular docking simulations were performed with AutoDock Vina 1.1.2 software,^44^ and molecular graphic representations were generated with UCSF Chimera^45^ and PyMOL 2.4.0 software.

### miRNA PCR Array Analysis

For the microRNA experiments, miRNA was extracted using the mirVana miRNA Isolation Kit (Qiagen, Germany). Then miRNA was converted to cDNA using the miScript II RT Kit (Qiagen, Germany), with 20 μL of the resulting cDNA diluted in 90 μL of RNase-free water applied to the 96-well miScript miRNA PCR array (MIHS-103ZA; Qiagen, Germany) containing primers for the detection of 84 associated miRNAs and duplicates of 6 internal controls. qRT–PCR was performed on a 7500 Real-Time PCR system (Applied Biosystems, USA), and the data were analyzed using GeneGlobe data analysis software (Qiagen, Germany). The PCR conditions for the miRNA array assay were 95 °C for 15 min, 94 °C for 15 s, 55 °C for 30 s, and 70 °C for 30 s for a total of 40 cycles. The best reference genes were selected using a web-based comprehensive tool (RefFinder) developed for evaluating and screening reference genes from extensive experimental data sets.^46^ The tool integrates the currently available major computational programs to compare and rank the tested candidate reference genes (geNorm, NormFinder, BestKeeper, and the comparative delta-Ct method).^47−49^ Based on the rankings from each program, it assigns an appropriate weight to an individual gene and calculates the geometric mean of their weights for the overall final ranking. microRNA quantification was evaluated based on the fold change and calculated using the 2^–ΔΔCT^ equation. SNORD95 and SNORD96A were selected as reference genes using RefFinder analysis.

### Bioinformatic Analysis

DIANA tools v.5.0 (http://diana.imis.athena-innovation.gr) was used to identify miRNAs associated with cell proliferation and cell death and related pathways. mirPath v.3 (https://dianalab.e-ce.uth.gr/html/mirpathv3/index.php?r=mirpath) was queried to identify miRNAs and their respective gene targets and signaling pathways based on the Kyoto Encyclopedia of Genes and Genomes (KEGG). Only signaling pathways with p values <0.05 were considered significant and further analyzed. For heatmap generation, the enrichment method of gene union/pathway union was used. STRING v.11.5 (https://string-db.org) was used to verify protein–protein interactions between the identified miRNA target genes. The network of the genes and selected miRNAs was constructed using Cytoscape v.3.9.1 (https://cytoscape.org/).

### Statistical Analyses

All data represent at least three independent experiments and are expressed as the mean ± standard error of the mean (S.E.M). Statistical analyses were performed using Student’s *t* test for comparisons between two groups and analysis of variance (ANOVA) and Dunnett’s post hoc test for multiple comparisons among groups. A probability value of *p* < 0.05 * vs the control was considered significant. GraphPad Prism 9.0.0 software was used.

## Supporting Information Available

Cell count and viability after 24 and 48 h; Dot plots and histogram from flow cytometry analysis of annexin V-FITC and 7-AAD staining and quantification of phospho-H2A.X, respectively; Functional network analysis of miRNAs; Unnormalized data of DNA-responsive proteins; Structure of 10-methoxy-canthin-6-one; Table of antibody description and conditions used (PDF)
