# Supplementary material for: DNA Damage-Inducing 10-Methoxy-canthin-6-one (Mtx-C) Promotes Cell Cycle Arrest in G2/M and Myeloid Differentiation of Acute Myeloid Leukemias and Leukemic Stem Cells
Source: ACS Omega. 2024 Aug 22;9(35):37343–54. doi: 10.1021/acsomega.4c05435 (PMC11375717; doi:10.1021/acsomega.4c05435)
Supplement: Supplementary file 1 — ao4c05435_si_001.pdf [file ao4c05435_si_001.pdf]

DNA damage-inducing 10-methoxy-canthin-6-one (Mtx-C) promotes cell cycle arrest in G_2/M and myeloid differentiation of acute myeloid leukemias and leukemic stem cells

Heron F. V. Torquato[1], Manoel Trindade Rodrigues Junior[4], Cauê Santos Lima[2], Roberto Theodoro de Araujo Júnior[2], Caio C. S. P. Soares[4], André Tarsis Domiciano[2], Rafael Leite Tavares de Morais[3], Daiane Rosolen[5], Luciane Regina Cavalli[5,6], Osvaldo Andrade Santos-Filho[7], Giselle Zenker Justo[2], Ronaldo Aloise Pilli[4], Edgar J. Paredes-Gamero[1,2*]*

[1]Faculdade de Ciências Farmacêuticas, Alimentos e Nutrição, Universidade Federal de Mato Grosso do Sul, 79070-900 Campo Grande, MS, Brazil

[2]Departamento de Bioquímica, Universidade Federal de São Paulo, R. Três de Maio 100, 04044-020 São Paulo, SP, Brazil

[3]Departamento de Biofísica, Universidade Federal de São Paulo, R. Três de Maio 100, 04044-020 São Paulo, SP, Brazil

[4]Instituto de Química, Universidade Estadual de Campinas, 13084-971 Campinas, SP, Brazil

[5]Instituto de Pesquisa Pelé Pequeno Príncipe, Curitiba, 80250-060, Brazil

[6]Lombardi Comprehensive Cancer Center, Department of Oncology, Georgetown University, Washington DC, 20007, USA

[7]Laboratório de Modelagem Molecular e Biologia Estrutural Computacional, Instituto de Pesquisas de Produtos Naturais Walter Mors, Centro de Ciências da Saúde, Universidade Federal do Rio de Janeiro, Av. Carlos Chagas Filho, 373 - Bloco H, Cidade Universitária, 21941-599, Rio de Janeiro, Brazil.

KEYWORDS. Natural Products, Alkaloids, Leukemia, Leukemic Stem Cells, Cell Cycle Arrest, Differentiation.

AUTHOR INFORMATION

*Corresponding author:
Heron F. V. Torquato
E-mail: heron.fvt@gmail.com
Faculdade de Ciências Farmacêuticas, Alimentos e Nutrição (FACFAN)
Laboratório de Biologia Molecular e Culturas Celulares
Av. Costa e Silva, s/n. Bairro Universitário. CEP: 79070-900
Campo Grande, MS, Brasil.
Tel.: +55 67 3345-7320

*Corresponding author:
Edgar J. Paredes-Gamero
E-mail: edgar.gamero@ufms.br
Faculdade de Ciências Farmacêuticas, Alimentos e Nutrição (FACFAN)
Laboratório de Biologia Molecular e Culturas Celulares
Av. Costa e Silva, s/n. Bairro Universitário. CEP: 79070-900
Campo Grande, MS, Brasil.
Tel.: +55 67 3345-7320

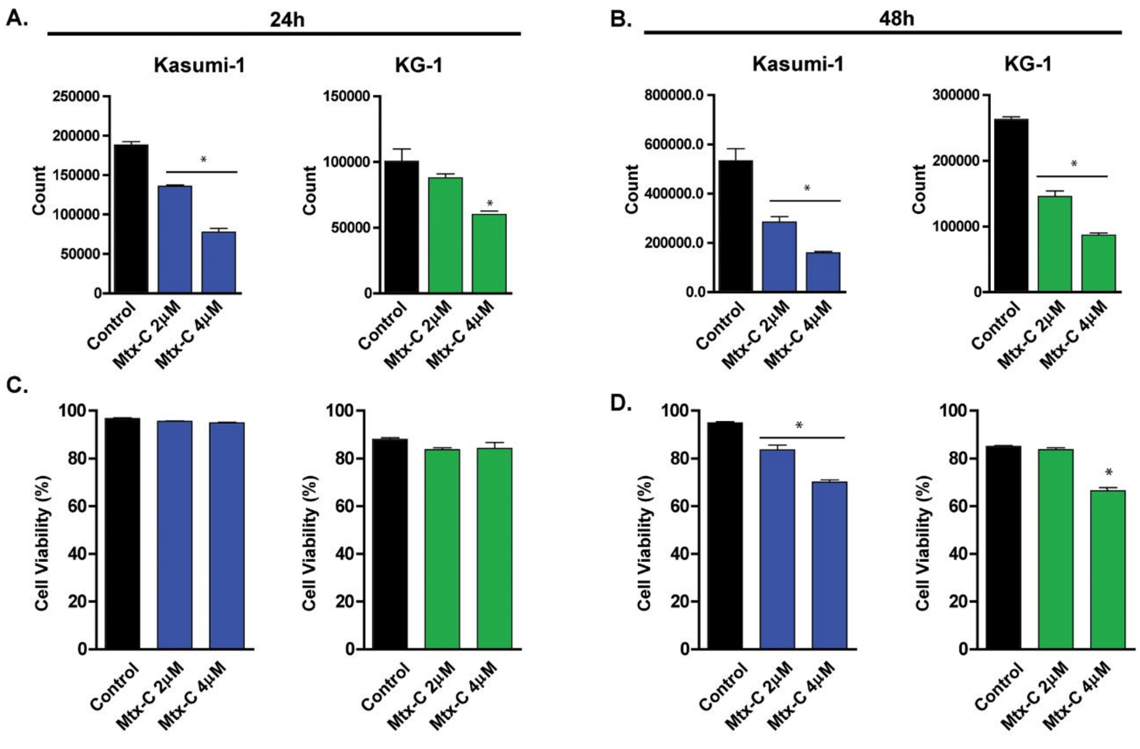

Figure S1. Human AML cell numbers and viability after Mtx-C treatment. The cells were treated once daily with 2 or 4 μM Mtx-C and incubated for 24 h or 48 h. The cells were counted after 24 h (A) and 48 h (B) with a flow cytometer. (C-D) Cell viability was measured by annexin V-FITC and 7-AAD staining. The data are presented as the means ± S.E.M.s. *p<0.05. One-way ANOVA followed by Dunnett's post hoc test.

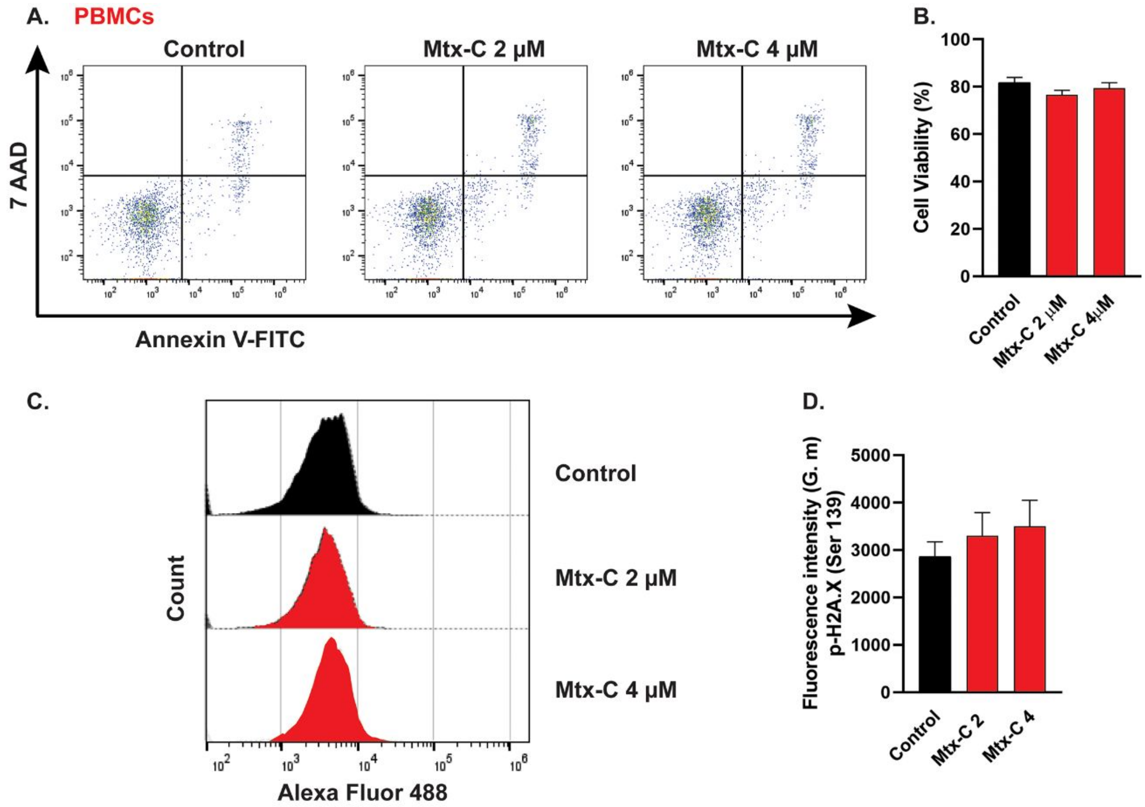

Figure S2. Mtx-C did not affect cell viability or the phosphorylation of H2A. X in PBMCs. The cells were incubated daily for 3 d with 2 or 4 μM Mtx-C. (A) Dot plots from flow cytometry analysis. (B) Cell viability was measured using annexin V-FITC and 7-AAD staining. (C) Histograms and (D) quantification of phospho-H2A.X. The data are presented as the means ± S.E.M.s. *$p<0.05$. One-way ANOVA followed by Dunnett's post hoc test.

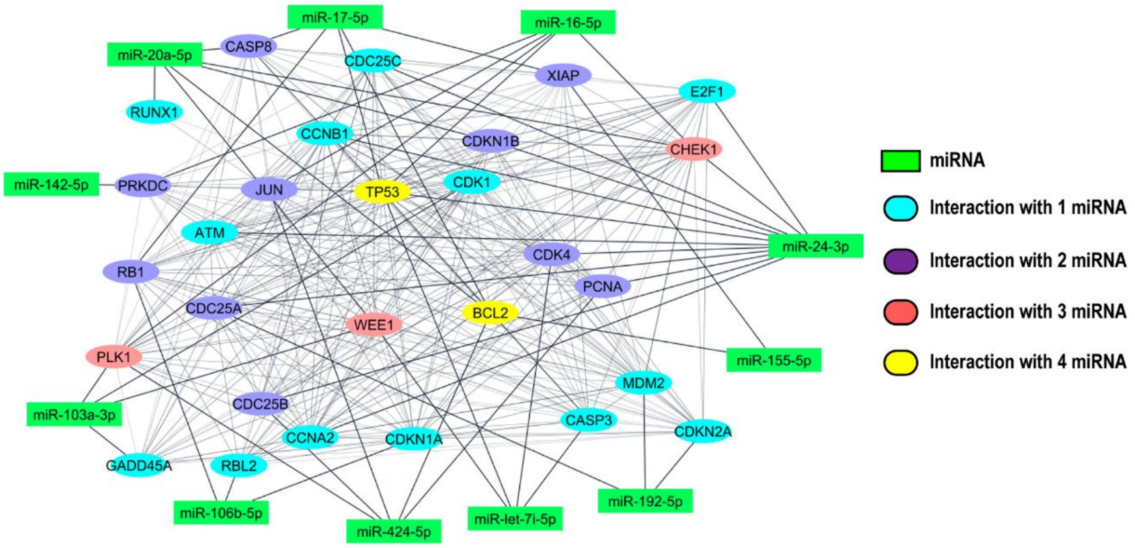

Figure S3. Functional network analysis of 11 miRNAs associated with AML cell proliferation or death after Mtx-C treatment. The figure illustrates the predicted interactions of miRNAs with their targets and the pathways associated with those target genes in Kasumi-1 cells.

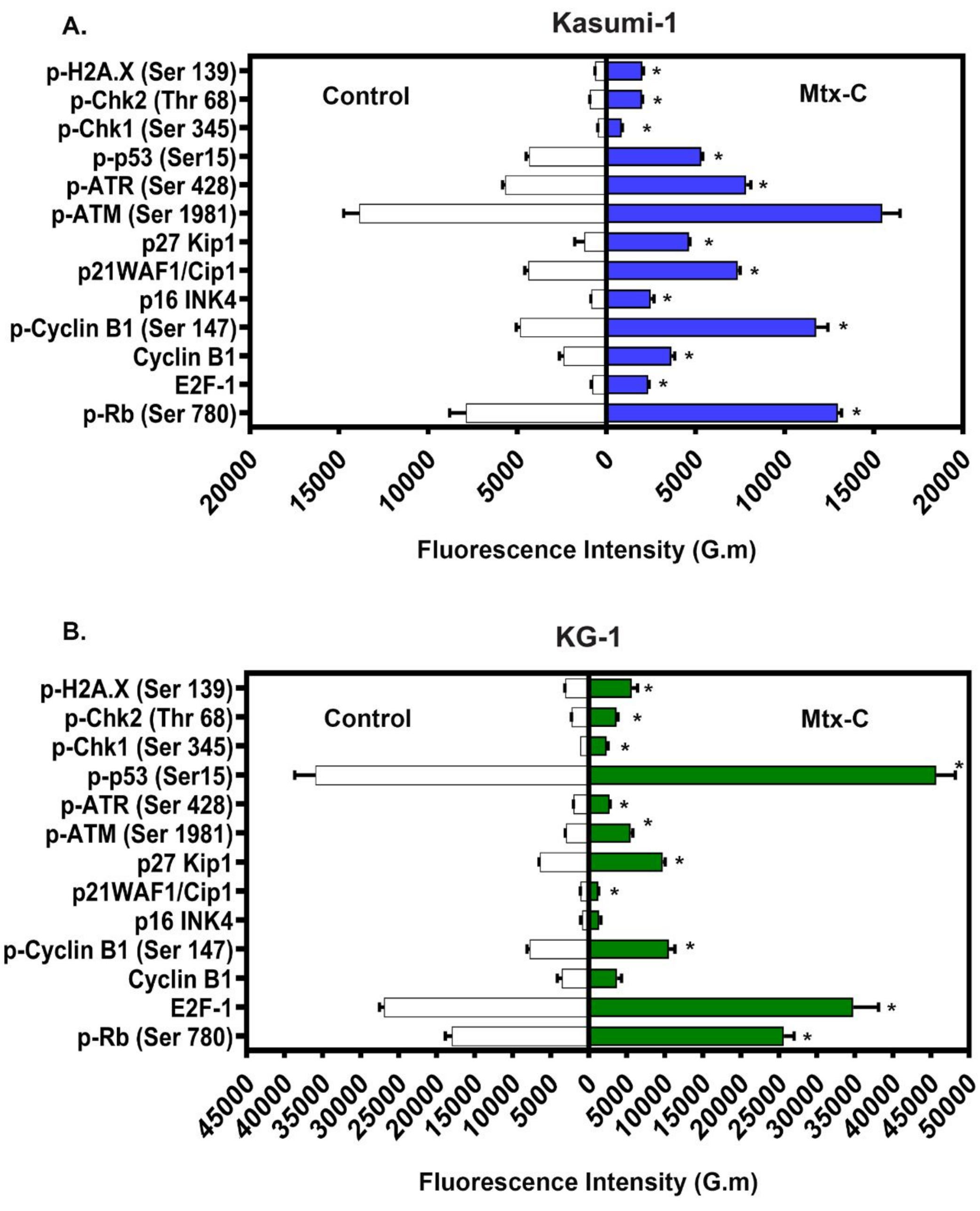

Figure S4. Unnormalized data presented with the geometric mean fluorescence intensity. (A) Kasumi-1. (B) KG-1. The results are presented as the means ± S.E.M.s of 3 independent experiments performed in triplicate. Student´s t-test. *p<0.05 versus control.

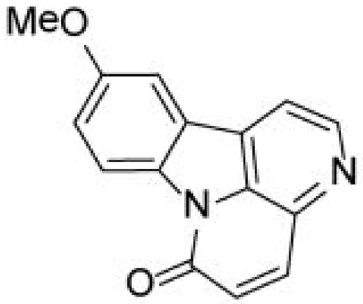

Figure S5. 10-methoxy-canthin-6-one

Table 1. Antibody description and conditions used.

Primary antibody	*Dilution*	*Isotype*	*Manufacturer*	*Secondary antibody*
p-p53 (Ser 15)	1:400	Mouse	C.Sign.#9286	AF 647
p.H2A.X (Ser 139)	1:400	Rabbit	C.Sign.#9718	AF 488
Ki-67 Alexa Fluor 488	5 μL	Mouse	BD Catalog 561165	-
Cyclin B1	1:200	Rabbit	C.Sign.#4138	AF 488
p-Rb (Ser 780)	1:100	Rabbit	C.Sign. #8180	AF 488
E2F-1	1:100	Rabbit	C.Sign.#3742	AF 488
p16^{INK4}	1:200	Rabbit	C.Sign.#80772	AF 488
p21$^{WAF1/Cip1}$	1:200	Rabbit	C.Sign.#2947	AF 647
p27 Kip1	1:400	Rabbit	C.Sign.#3686	AF 488
p-ATM (Ser 1981)	1:100	Rabbit	C.Sign.#5883	AF 488
p-ATR (Ser 428)	1:100	Rabbit	C.Sign.#2853	AF 488
p-Chk1 (Ser345)	1:100	Rabbit	C.Sign.#2348	AF 488
p-Chk2 (Thr 68)	1:200	Rabbit	C.Sign.#2197	AF 488
PU.1	1:200	Rabbit	C.Sign.#2258	AF 488
p-p38 (Thr 180/Tyr 182)	1:100	Rabbit	C.Sign.#9215	AF 488
p-Cyclin (Ser 147)	1:200	Rabbit	C.Sign.#4131	AF 488

C.Sign. (Cell Signaling); BD (Becton Dickinson); AF (Alexa Fluor)
Secondary Antibody Dilutions: AF 488 – 1:1000; AF 647 – 1:800
